# Attitudes, norms and controls influencing lifestyle risk factor management in general practice

**DOI:** 10.1186/1471-2296-10-59

**Published:** 2009-08-26

**Authors:** Amanda J Ampt, Cheryl Amoroso, Mark F Harris, Suzanne H McKenzie, Vanessa K Rose, Jane R Taggart

**Affiliations:** 1 Centre for Primary Health Care and Equity, University of New South Wales, Sydney, NSW, Australia; 2 Rural Clinical School (Sydney Campus), University of New South Wales, Sydney Australia; 3 School of Public Health & Community Medicine, University of New South Wales, Sydney, NSW, Australia; 4 School of Biomedical and Health Sciences, University of Western Sydney, Campbelltown, NSW Australia

## Abstract

**Background:**

With increasing rates of chronic disease associated with lifestyle behavioural risk factors, there is urgent need for intervention strategies in primary health care. Currently there is a gap in the knowledge of factors that influence the delivery of preventive strategies by General Practitioners (GPs) around interventions for smoking, nutrition, alcohol consumption and physical activity (SNAP). This qualitative study explores the delivery of lifestyle behavioural risk factor screening and management by GPs within a 45–49 year old health check consultation. The aims of this research are to identify the influences affecting GPs' choosing to screen and choosing to manage SNAP lifestyle risk factors, as well as identify influences on screening and management when multiple SNAP factors exist.

**Methods:**

A total of 29 audio-taped interviews were conducted with 15 GPs and one practice nurse over two stages. Transcripts from the interviews were thematically analysed, and a model of influencing factors on preventive care behaviour was developed using the Theory of Planned Behaviour as a structural framework.

**Results:**

GPs felt that assessing smoking status was straightforward, however some found assessing alcohol intake only possible during a formal health check. Diet and physical activity were often inferred from appearance, only being assessed if the patient was overweight. The frequency and thoroughness of assessment were influenced by the GPs' personal interests and perceived congruence with their role, the level of risk to the patient, the capacity of the practice and availability of time. All GPs considered advising and educating patients part of their professional responsibility. However their attempts to motivate patients were influenced by perceptions of their own effectiveness, with smoking causing the most frustration. Active follow-up and referral of patients appeared to depend on the GPs' orientation to preventive care, the patient's motivation, and cost and accessibility of services to patients.

**Conclusion:**

General practitioner attitudes, normative influences from both patients and the profession, and perceived external control factors (time, cost, availability and practice capacity) all influence management of behavioural risk factors. Provider education, community awareness raising, support and capacity building may improve the uptake of lifestyle modification interventions.

## Background

Australia, like other developed countries, is experiencing the associated social and economic burdens that arise from increasing rates of chronic disease. Seventy-seven percent of the Australian population had at least one long-term condition during 2004–2005, with 60% of the country's total health expenditure allocated to long-term conditions during 2000–2001 [1]. The impact on primary health care is substantial, with seven out of ten general practice (family physician) encounters being for chronic conditions [2].

The behavioural risk factors of smoking, nutrition, alcohol, and lack of physical activity **(SNAP) **are responsible for a substantial portion of chronic disease [3]. It is estimated that approximately 17% of Australians are tobacco smokers and 51% do not obtain enough exercise. Only 30% of males and 36% of females consume sufficient vegetables each day, and 48% and 60% respectively consume enough fruit [4]. These findings are alarming, especially in the context of the current burden of chronic disease in the Australian community.

The focus on preventing chronic disease is increasing in Australia. This interest is reflected in The Council of Australian Governments' Plan for Better Health for All Australians [5], and in the National Chronic Disease Strategy [6], both of which identify the importance of promoting healthy lifestyles by addressing risk factors in general practice. In order to support the aim of "prevention of chronic disease...enable early intervention strategies to be put in place where appropriate" [7], the 45–49 year old health check was introduced as a Medicare item in Australia on November 2006 [8]. The health check allows for a rebate to be paid to general practitioners (GPs) who deliver a preventive health consultation (with emphasis on SNAP factors) to a patient aged 45–49 years of age with at least one chronic disease risk factor (lifestyle, biomedical or family history). It is the first rebateable item specifically targeting chronic disease prevention, and delivery by the GP includes taking a patient history with relevant clinical examination and investigations, assessing patients' readiness to make appropriate lifestyle changes, initiating referrals and interventions, and providing advice and information [7]. Various templates have been designed to assist GPs in this process, however their use is determined by GP individual choice. Patients can be approached by opportunistic screening when they present for other consultations, or by systematic recall depending on the practice procedures and GPs' preferences. Initial uptake of this item has been documented in its first year [9]. Early research demonstrates some patient improvement in diet and physical activity, and describes the implementation of the health check [10]. GPs varied in their number of consultations, with the majority delivering the item over two to three sessions.

There is currently a gap in the knowledge of factors that influence the delivery of lifestyle risk factor screening and management by GPs [11]. Given the prevalence of the SNAP risk factors within the Australian community, GPs need to prioritise which patients receive risk factor assessment and management. Guidelines pertaining to behavioural risk factor screening and management are generally less prescriptive than other areas of medical practice, with more room for individual GP judgement. The introduction of the 45–49 year old health check provided the opportunity for research into GPs' decision-making around these issues. This qualitative study addressed the following specific research questions:

1. What are the factors that influence GPs' choosing to opportunistically screen for some, but not all, SNAP risk factors in a health check?

2. What are the factors that influence GPs' choosing to provide interventions for some, but not all, SNAP risk factors when present in a patient who is at risk for chronic disease?

3. When multiple SNAP risk factors are present, what factors influence the decision regarding which risk factors to address and in what order?

Knowledge of these key influences may increase the effectiveness of future chronic disease interventions, with the ultimate aim of informing practice to promote more preventive care.

## Methods

Audio-taped interviews were conducted over two stages with 15 GPs and one practice nurse from two geographical areas in Sydney Australia, by two researchers (AA and CA). The majority of GPs had been involved in a recent study examining the feasibility and impact on practices and patient behaviour of the 45–49 year old health check, during which patients were specifically recruited for involvement [10]. The initial semi-structured interview was piloted with another GP who was not otherwise involved in the study, and minor adjustments were made to the wording and sequencing of some interview questions. This initial interview explored the introduction of the 45–49 year old health check in general, as well as reflection regarding specific consultations regarding SNAP assessment and management practices. Interviews were undertaken until data saturation was reached. During the second stage of the interviews (after initial analysis and preliminary model development had been undertaken) findings and interpretations were discussed with the clinicians, during which clinician feedback and further clarification regarding the data analysis were sought in a process described as "communicative validation" [12].

Clarification and further development of themes were incorporated into the refinement of the subsequent model. More details regarding individual SNAP factors were also sought which assisted in researchers' interpretation of data.

All interviews were conducted in the GPs' rooms during 2007. Ethical approval was granted by the University of New South Wales Human Research Ethics Committee. Participants were given written information sheets, signed a written consent prior to being interviewed, and all information obtained in the interviews was de-identified.

### Analysis

The de-identified interview transcripts were transcribed by an external typing service. The transcriptions were then read by the interviewing researchers while listening to the audio files of the interviews in order to check and confirm accuracy. Analysis was undertaken with the assistance of QSR NVivo7 software. Emerging themes were identified and a coding framework was developed by both interviewing researchers in collaboration with each other. Analysis continued with constant comparison and further refinement of coding, with one researcher undertaking the coding and the other checking the analysis. Any discrepancies in interpretation or coding were discussed, with feedback from the other researchers sought.

The Theory of Planned Behaviour [13] was used as an overarching structural framework to support the final analysis, and to develop a model of factors influencing preventive care in the health check consultation. Its adoption for this research was influenced by its relevance to analysing factors affecting current behaviour at the clinician level, and not the organisational level. Other theoretical models that have been applied to preventive care were considered, but rejected on the basis of either applying predominantly to levels of clinician confidence rather than actual behaviour (e.g. those based on the Theory of Self-Efficacy); of providing a framework for intervention rather than performance (e.g. the Transtheoretical Model of Change); or of analysing adoption of innovation (e.g. Rogers Diffusion of Innovation Theory). The Theory of Planned Behaviour was useful in guiding identification of both external and internal factors that specifically influenced clinician behaviour. The final adaptation of this theory to a model was refined after feedback from other members of the research team and from clinicians during the second interview.

**Figure 1:**
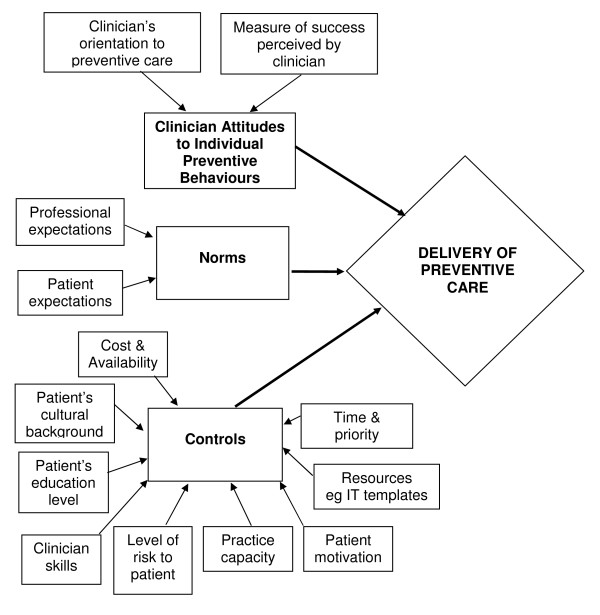
**Model: The Theory Of Planned Behaviour as applied to preventive care delivery in general practice**.

The Theory of Planned Behaviour states that the performance of a behaviour is influenced by attitudes, by norms, and by hindering or facilitating controls. Attitudes reflect the "degree to which a person has a favourable or unfavourable evaluation of the behaviour in question"; norms reflect the perceived social pressures to perform or not perform the behaviour; and controls reveal the perceived ease or difficulty of performing the behaviour [13]. The identified themes from this study articulated well into this theory, from which a model of delivery of preventive care in general practice was developed. This model is presented in Figure 1, and summarises the results presented below.

## Results

### Characteristics of the sample of GPs

The 15 GPs in this sample consisted of seven males and eight females, representing an approximately equal number of single and group practices. The majority had previously been involved as participants in a related study [10], and had undertaken a short training session in motivational interviewing. During the recruitment process, these GPs had expressed an interest in the project. It is therefore recognised that this sample represents GPs who may be more interested in preventive care, or more specifically interested in identifying the barriers and enablers to delivering such care, than the general population of GPs.

### Behaviours of GPs

The provision of preventive care in a health check consultation involves a number of possible behaviours by clinicians. Initial open coding of the interview transcripts assisted in the identification of the various behaviours. Not all behaviours were undertaken by all clinicians, and clinicians discussed the execution of them to different degrees. Added to that, some clinician behaviours were more evident for some SNAP factors than for others. Further analysis of the transcripts allowed the researchers to identify specific factors influencing these behaviours. These are presented in the following section, with supporting quotes presented in the appendix.

### Assessment (relates to research question 1)

The assessment of lifestyle risk by clinicians included direct enquiry, ordering tests, and reviewing and updating patient records. Some GPs stated that patients expected risk factors to be assessed within a health check consultation, thus providing a sense of greater permission to address SNAP factors. Time was mentioned as a barrier to a detailed assessment. Differences in assessment for individual SNAP factors were also evident. GPs were unanimous in their opinion that reviewing smoking status was straightforward. There was more variation in their attitudes towards assessment of nutrition, alcohol and physical activity. Those GPs who had experience and interest in addressing drug and alcohol issues reported being consistent in assessing alcohol intake; others had increased this screening as a result of implementing the health check, and some others felt this screening was only possible during such a health check. Nutritional status and level of physical activity were often inferred by the clinicians from the patient's general appearance (eg overweight), or from physiological conditions such as hypertension or hypercholesterolaemia. The level of risk to the patient appeared to inform the intensity of the assessment. For example, if the patient already exhibited signs of poor nutrition (such as obesity), more intensive assessment of diet and physical activity would usually be undertaken.

The GPs' perception of their professional role also influenced the amount of assessment, with one GP admitting to not asking about specific dietary intake as he "was not a dietician" and doubted the effectiveness of general dietary recommendations.

Those GPs who did fully assess nutrition, or specifically asked about physical activity, were influenced by other factors. These included the capacity of the practice (eg a nurse who undertook assessments), or the expressed interest of the GP in these risk factors. Specific mention was also made of the usefulness of a computer-based template or patient education and assessment resources such as Lifescripts, which include paper-based templates for lifestyle assessment and individualised written prescriptions for behaviour change including goals and actions [14].

### Motivating the patient (relates to research question 2)

GPs varied in their attempts to motivate their patients to change risk behaviour. This was discussed in the wider context of how much preventive care they were involved in generally, whether they felt effective as a motivator, and whether it was an expected role of GPs. Most had recently attended training in motivational interviewing, with some GPs believing their skills had increased. Others felt that motivational interviewing sounded good in theory, but the reality of practice demands made it difficult. Some expressed disappointment when they could not successfully motivate their patients, implying that this was part of their professional role. At the opposite end of the spectrum, others felt that once the patient had been educated regarding lifestyle risk factors, the responsibility then lay fully with the patient. The patient's intrinsic level of motivation was often discussed, rather than whether the GP could modify that level.

Motivating patients to stop smoking caused the most frustration. One GP stated he no longer attempted it, while others believed they just did not have the skills to succeed. Success was generally gauged by the patient quitting completely. Success was similarly judged for patients who engaged in heavy drinking, with abstinence as the goal. This was different from the other lifestyle factors where success was located on a continuum from incremental improvement to sustained achievement of dietary and/or exercise goals. General practitioners who recognized that success for weight reduction could include small weight losses voiced less frustration than those whose measure of success was the achievement of ideal weight goals. Some GPs also reported that a patient's education level influenced the motivation level of that patient.

### Giving Advice and educating the patient (relates to research question 2)

Giving advice and educating the patient were viewed as a professional responsibility by all GPs, and reported as expected by the patients. How the advice was given varied considerably amongst the clinicians, from personalized negotiation with patients to didactic presentation. When GPs recognized cultural influences on dietary habits, they tried to incorporate those cultural aspects into their advice. Whether patients were given written information depended more on clinician preferences than patient choice. The majority of GPs felt printed material reinforced any message. Written information appeared more commonly used to support nutritional advice followed by physical activity. Written materials for alcohol and smoking cessation were reported as being used less often. Some GPs felt they lacked skills in the area of nutrition, and wished they had better knowledge. This may have influenced their choice in giving written information to their patients.

The amount of diet and physical activity advice was proportional to patient risk (such as having an identified weight problem).

### Arranging follow-up appointments (relates to research question 2)

There was recognition that ongoing behavioural change usually required more support than a single visit, but had to be balanced with the reality of practice demands. The degree to which follow-up appointments were encouraged appeared to depend upon the GPs' orientation to preventive care in general. Different attitudes were also evident in practices that had an appointment system (compared with a 'drop-in' service) as follow-ups could more easily be arranged. Follow-up was also provided opportunistically when patients came in for other reasons. Smoking and alcohol were followed up more actively, while nutrition and physical activity were followed up only if the patient was already overweight or hypertensive.

The patient's level of motivation was often cited as an influencing factor. In addition, cost was a perceived barrier for patients to return to the surgery, with many GPs believing that patients were reluctant to pay for ongoing consultations regarding preventive care.

### Referring to other personnel and agencies (relates to research question 2)

GPs exhibited a range of attitudes towards referring patients to other services and personnel, with some believing that health conditions should be managed predominantly by the GP. GPs appear to be more ready to refer for some SNAP risk factors than others, with smoking prompting the most enthusiasm for referral. This was consistent with the frustration many GPs experienced with smoking cessation.

Perceived patient resistance to out of pocket costs was often cited as a barrier to referral, with many GPs believing their patients would not take up referrals if they had to pay. The accessibility of services was also important. With the majority of 45–49 year olds being in employment, most found it difficult to access referrals in working hours, and many services were not available out of hours. Quitline (a free smoking referral telephone service) does not have these barriers and was by far the most common referral pathway.

Patient motivation was also mentioned as a barrier. Only one GP acknowledged that support services could actually help to motivate patients.

Referrals to dieticians for nutritional advice appeared to depend on the patient's level of risk. Some GPs felt the advice offered by a dietician would be no different to that offered by the GP, and thus referrals were of little value.

Many GPs voiced their lack of knowledge regarding the role of the exercise physiologist. One GP stated that she referred to physiotherapists because she had worked with them in hospitals, but had no idea what an exercise physiologist did. Without this knowledge, she was reluctant to refer as she could not vouch for their effectiveness. Referrals to gyms and exercise classes were considered by GPs, but concern was expressed about the cost to the patient.

Referral to self help groups such as Alcoholics Anonymous was rarely mentioned as few GPs felt their patients were drinking at levels which required this level of intervention.

### Managing multiple SNAP factors (relates to research question 3)

When clinicians were presented with a hypothetical patient with multiple risk factors, and asked how they would proceed with intervention, there was a variety of responses reflecting a non-standardised approach. Some GPs mentioned that they would try and motivate patients to address alcohol problems first, especially if they were drinking at levels high enough to de-stabilize their lives in relation to work and relationships – in other words, if the level of risk was high. One GP mentioned that if the patient was educated, they would try and address all the SNAP factors in one go. Others focused on assisting the patient to deal with whichever risk factor the patient wished to tackle first as this had a greater chance of success.

This view that the intervention should be patient-led was the most common, with a consensus that potential greater success may be achieved by addressing whichever factor the patient was more ready to change first. If the patient was unsure how to proceed, and appeared motivated to address all factors, smoking was often considered first. Other GPs felt it might be more advantageous to start with diet and/or physical activity, as changes in these areas would result in the patient feeling better more quickly. One clinician mentioned that if all SNAP factors were present, a psychological assessment should be made first.

The method of addressing individual factors when multiple factors were present followed the same variety of responses as outlined previously, with behaviours reflecting a range of strategies.

## Discussion

It is recognised that the findings from this self-selected group may not be broadly transferable to the general population of GPs; however they do provide insight into the barriers and enablers that GPs face in delivering preventive care within a health check.

The performance of preventive care by GPs within the 45–49 year old health check was influenced by attitudes, by norms or expectations, and by controls. Attitudes included the level of clinicians' belief that a certain behaviour would produce a desired outcome, and incorporated the clinicians' feelings about their own effectiveness in ultimately promoting lifestyle behavioural change in patients – the end result of the preventive care behaviour. This is consistent with research in Europe where GPs have expressed concern about the effectiveness of lifestyle interventions [15]. Attitudes were strongly influenced by the clinicians' general orientation to the importance of preventive care, as well as how they measured a successful outcome. In addition, attitudes influenced referral to other agencies, as belief (or lack of it) in the potential effectiveness of the service was a key element in referring behaviours.

Norms included the expectations of the professional community. Implicit in these expectations were the GPs' self perceptions of their role in preventive care behaviour. These perceptions varied amongst the GPs, especially with regard to motivating the patient and providing referrals. However most GPs felt that lifestyle interventions were a core part of their role. This accords with studies overseas [16]. The expectations of the patient community also influenced behaviour. GPs expressed the opinion that patients expected lifestyle factors to be raised within a health check. This may be different from other consultations, providing added incentive for preventive care.

Controls included those factors that facilitated and hindered the actual preventive care behaviours. Examples of facilitating controls as revealed by the GPs included increased level of risk to the patient, provision of computer-based templates, motivated and educated patients, good clinician skills and knowledge, reasonable practice capacity in terms of personnel and time, and low cost and good availability of referral sources. Financial remuneration was rarely raised as an influencing factor.

## Conclusion

In asking GPs to address SNAP lifestyle factors through the 45–49 year old Health Check consultation, it is important to understand how GPs view their role in preventive care, and in addressing the individual SNAP factors. This study has focused questions around these influences and behaviours, with the aim of better enabling GPs to provide such care. It is recognized that the participating GPs self-selected, and as such may not be representative of the wider GP community who may be less oriented to preventive care.

Given the complexity of factors influencing GPs' preventive behaviour, it is not surprising that their approaches varied across the individual SNAP factors, and between different patient groups. However within this variety, commonalities did exist. For example smoking appeared to universally provoke the most frustration. Another commonality was the trend for patients to be only partially screened for nutrition and physical activity, with assessment frequently inferred from or triggered by physiological markers such as weight, cholesterol levels and blood pressure.

This study provides some insights that will be important in the development and implementation of preventive activities in general practice. GPs knowledge and attitudes are important factors. However norms and control factors also need to be addressed. This has been observed in other studies [17]. Strategies such as provider education, community awareness raising, funding support and capacity building may potentially be able to improve both the uptake of health checks and ensure a more consistent approach. However this study also demonstrates the variability between GPs, and the importance of adapting the approach of management of lifestyle risk factors to the practice and patient population. Changing GP attitudes towards referral services is likely to take more prolonged interventions and more direct experience of their effectiveness.

## Competing interests

The authors declare that they have no competing interests.

## Authors' contributions

AJA undertook interviews, thematic coding and analysis; assisted in study coordination and prepared the manuscript. CA participated in the conception and design of the study; undertook interviews, thematic coding and analysis; coordinated the study and helped draft the manuscript. MFH conceived the study and participated in its design, participated in the analysis and helped draft the manuscript. SMcK participated in the design of the study, its analysis and helped draft the manuscript. VR participated in the design of the study, its analysis and helped draft the manuscript. JT participated in the design of the study, its analysis and helped draft the manuscript. All authors read and approved the final manuscript.

## Appendix: Examples of Supporting Quotes Relating to Identified Preventive Care Behaviours

### Assessment

... in a normal consult, I don't raise all of these risk factor issues with them so I can't really compare but during the health checks they were quite acceptable

*Rather than what they're eating, I ask about nutrition only if the weight is very high and if they (are) obviously well looking person, I don't bother*.

The only thing I wouldn't have picked up is nutrition and physical activity – but now it does as it forces it to be assessed through the template

### Motivating the Patient

*...now you can address some of the risk factors and assess somebody's willingness to make changes in their lifestyle and with the particular point they're up to so you know whether or not you're wasting your time by going ranting and raving..*.

*We are not here as saints to – I mean we need to move on with our time and there are some ten other patients for one unmotivated patient who we can help so if in the end the patient's not motivated I think motivational interviewing is not going to make a huge difference*.

*... I think it's very, very difficult to motivate patients give up smoking ...sometimes I think it's a waste of time from my part ..*.

*I cannot expect him to be 25 BMI. It's never going to be possible ... if he changes just a few things and he maintains his weight or it doesn't increase more that would be my success or if he manages to lose even 5 kg and keeps a rapport with me ... I think we've done him something ..*.

### Giving Advice and Educating the Patient

*I was saying "you've got a weight problem and obviously the cholesterol is raised, you need to perhaps lose some weight ... eat healthily, eat more vegetables and to exercise regularly, all you need to do is just start walking and do it regularly". So basically I gave her information*.

*Everybody is different. You've just got to find the right formula that clicks one person in the right direction rather than another*.

### Arranging Follow-Up Appointments

*...I did try to practice preventative medicine when you see obese and if they've got diabetes or heart disease, of course I – but I must say it's not structured with the timeframe allowed 'cause this is about 20 minutes or more ...and that's a bit hard sometimes to spare 20 minutes*.

It's very difficult and it's not the money, it's the time, the logistics to see all these patients... How many long consultations can you give?

### Referring to other personnel and agencies

*My philosophy's in-house as far as possible on any medical condition. I'm not a high referral source..*.

...because if I'm going to refer them and if they are not quite motivated and then it's going to fail

*Okay if the patient has high cholesterol or hypertensive and perhaps is overweight we'll discuss their diet and that sort of things and I would ask them if they would like to see a dietician*.

### Managing multiple SNAP factors

*I'd try to sort out what the patient had in mind, maybe he or she wants to do the lot all together as a package, we'd have to come to some agreement as to how the patient wants to address it. But it needs to be patient-orientated where possible*.

## Pre-publication history

The pre-publication history for this paper can be accessed here:


